# Aqueous microdroplets containing only ketones or aldehydes undergo Dakin and Baeyer–Villiger reactions†
†Electronic supplementary information (ESI) available. See DOI: 10.1039/c9sc05112k


**DOI:** 10.1039/c9sc05112k

**Published:** 2019-11-12

**Authors:** Dan Gao, Feng Jin, Jae Kyoo Lee, Richard N. Zare

**Affiliations:** a Department of Chemistry , Stanford University , Stanford , CA 94305 , USA . Email: zare@stanford.edu; b State Key Laboratory of Chemical Oncogenomics , Tsinghua Shenzhen International Graduate School , Tsinghua University , Shenzhen , 518055 , China; c Shenzhen Deepdrug Information Technology Co. Ltd. , Shenzhen , 518052 , China

## Abstract

The Dakin and Baeyer–Villiger (BV) oxidation reactions require addition of peroxides as oxidants and an acid or a base as a catalyst. Reaction times range from hours to days to obtain target products. Previously, we reported that hydrogen peroxide (H_2_O_2_) is spontaneously generated in water microdroplets without any added chemicals or applied electrical potential. Here, we report that the Dakin and BV reactions occur in modest yields within milliseconds in aqueous microdroplets at room-temperature without the addition of external peroxides and catalysts. H_2_O_2_ generation is the result of the special environment of the microdroplet surface, which promotes water autoionization. We find that increasing the content of water and decreasing the droplet size improve the product yield of the Dakin and BV reactions, supporting the contention that the amount of H_2_O_2_ generated in aqueous microdroplets could induce the two reactions and the reactions occur at or near the air–water interface of the microdroplet surface.

## Abstract 2

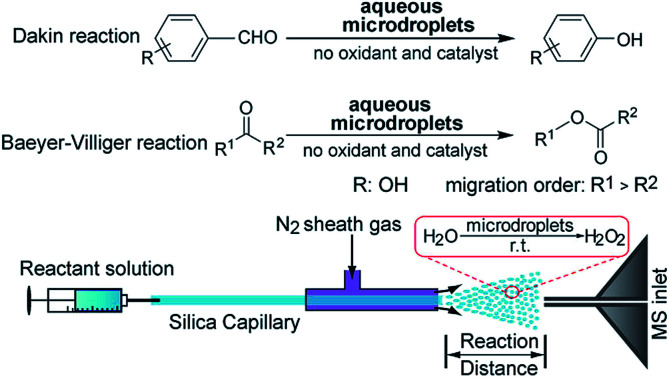
The Dakin and Baeyer–Villiger (BV) oxidation reactions require addition of peroxides as oxidants and an acid or a base as a catalyst.

## Introduction

Many studies have shown that the rates of chemical reactions in microdroplets sprayed in air under ambient conditions can be markedly accelerated compared to the same reactions in bulk solution.^1^ It is also possible for the reaction products in microdroplets to differ substantially compared to those from the same reaction conditions in bulk solution.^2^ Recent work has demonstrated that aqueous microdroplets can cause spontaneous reduction. Two examples are the formation of gold nanoparticles and nanowires from aqueous microdroplets containing only chloroauric acid,^3^ and the reduction of a number of different organic compounds dissolved in aqueous microdroplets, such as the conversion of pyruvate to lactate.^4^ In all these cases, no external potential is applied to the spraying source for microdroplet generation and the room-temperature aqueous microdroplets do not contain other compounds, such as catalysts. Perhaps, the most striking example is the recent discovery that aqueous microdroplets under ambient conditions (room temperature and 1 atmosphere pressure of air) contain a small concentration (∼30 μM) of hydrogen peroxide (H_2_O_2_).^5^ In this study, we demonstrate that the H_2_O_2_ formed in water microdroplets can be used to carry out the Dakin and Baeyer–Villiger (BV) reactions, which are classic synthetic approaches for the oxidation of carbonyl groups.

Dakin oxidation is one of the most common chemical reactions for the synthesis of phenols from hydroxybenzaldehydes, and BV oxidation is often used to prepare esters from ketones.^6^ Both reactions need aldehydes or ketones and peroxides (typically hydrogen peroxide (H_2_O_2_) or peroxyacid) as reactants with the addition of acid or base (such as NaOH) catalysts.^7^ Most modern synthetic strategies for these oxidation reactions require hazardous peroxides, a high concentration of acid or base or an expensive organometallic compound as the catalyst. Moreover, the Dakin and BV reactions typically need hours to days to obtain target products.^8^ Therefore, many efforts have been made to develop oxidation systems with aqueous H_2_O_2_ as the oxidant^9^ or biocatalytic reagents such as BV monooxygenases.^10^

**Fig. 1:**
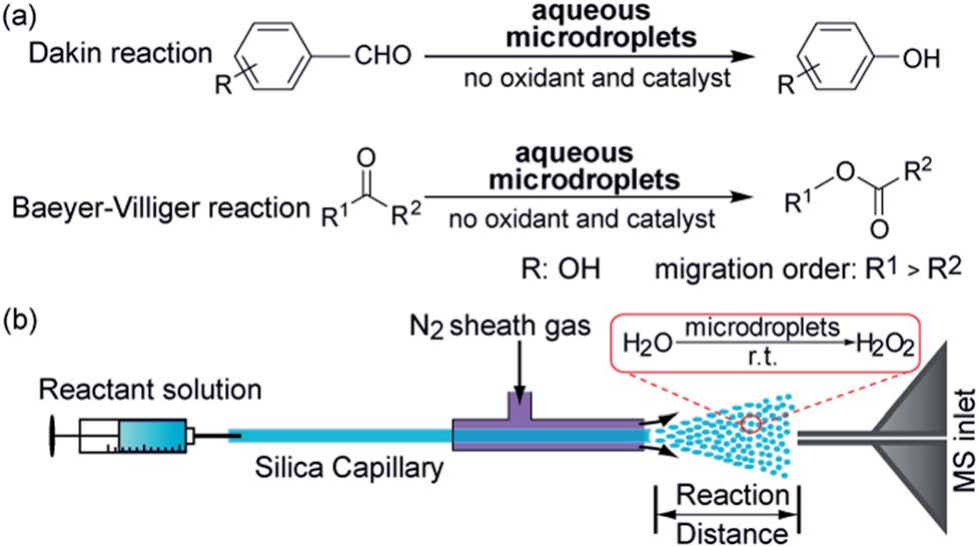
(a) Dakin oxidation in microdroplets of hydroxybenzaldehydes to phenols and BV oxidation of ketones to esters (R^1^ and R^2^ denote the migrating groups). (b) Schematic of the experimental setup used to run and monitor the Dakin and BV reactions in aqueous microdroplets sprayed in air.

Due to the unique characteristics of aqueous microdroplets, like production of H_2_O_2_ and hydrated excess hydronium and hydroxide ions on the microdroplet surface,^11^ we present a proof of principle here that the Dakin and BV reactions can be achieved using aqueous microdroplets containing only the carbonyl compound that is desired to be transformed (see Fig. 1a). Much more work needs to be done to show how such reactions can be scaled up and how yields can be improved to make this method practical for preparative synthesis. It is hoped that this work is a first step in realizing this goal.

## Results and discussion

Fig. 1b shows our experimental setup. A 1 : 1 solution of water and acetonitrile (to aid solubility) containing aldehyde or ketone was sprayed with a high-pressure nebulizing gas (N_2_, 120 psi) to produce microdroplets encapsulating the reactants with sizes ranging from 1 to 50 μm in diameter.^12^

**Fig. 2:**
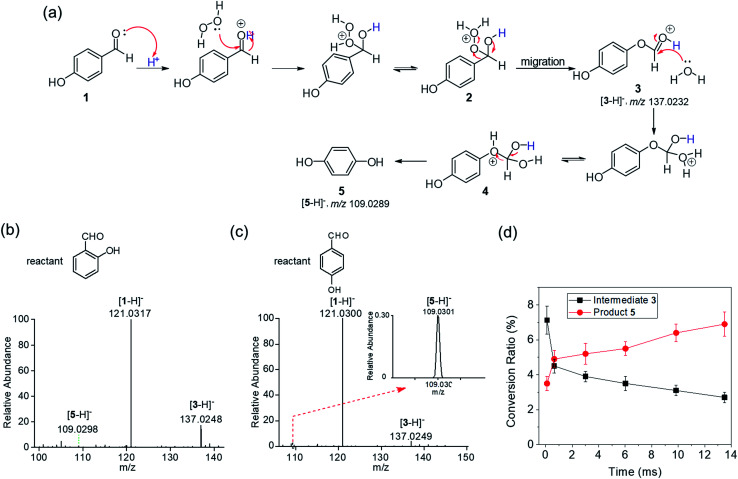
The Dakin reaction of *ortho*- or *para*-hydroxybenzaldehyde in microdroplets: (a) reaction scheme; (b) and (c) mass spectra using *ortho*- and *para*-hydroxybenzaldehyde; and (d) kinetics of intermediate **3** and product **5** ranged from microsecond to millisecond timescale by adjusting the distance between the spray emitter to the MS inlet.

The Dakin reaction is an oxidation reaction in which hydroxybenzaldehyde is converted to a phenol on treatment with H_2_O_2_ (Fig. 2a). The classical mechanism of the Dakin reaction includes the following steps. 4-hydroxybenzaldehye **1** is protonated followed by the attack of hydrogen peroxide on the carbonyl carbon to form a tetrahedral intermediate **2**. This step is followed by aryl migration and water elimination to yield a phenyl ester **3**. Then hydrolysis of **3***via* intermediate **4** yields the final product **5**, 1,2-dihydroxybenzene. We dissolved *ortho*- or *para*-hydroxybenzaldehyde in water/acetonitrile (1 : 1, v/v) and sprayed this solution using our experimental setup into the inlet of a mass spectrometer for real-time analysis of reaction products. As shown in Fig. 2b and c, we observed a small amount of *ortho*- or *para*-hydroxyphenol **5** in microdroplets containing reactant **1**, hydroxybenzaldehye. We also observed the expected intermediate **3** in the two Dakin reactions. The chemical structures of intermediate **3** and product **5** were confirmed by comparing observed fragment ions from the microdroplet reactions and the corresponding standard samples with tandem mass spectrometry using collision induced dissociation (CID) (see Fig. S1 and S2†). The observation of intermediate **3** supports the above-indicated reaction mechanism. In the bulk phase, the Dakin reaction is carried out with excess H_2_O_2_ and high concentrations of acid or base at elevated temperatures.^13^ This means that the reaction cannot occur when only the reactant is present in bulk water–organic solvents at room temperature. However, our results indicated that the amount of H_2_O_2_ generated^5^ (as well as the hydrated excess hydronium and hydroxide ions on the surface of the microdroplets^11^ could meet the requirement of oxidants as reactants and drive acid/base-catalyzed Dakin reactions. It might seem paradoxical that both the oxidation reaction studied in this work and the reduction reaction reported by our previous work can occur in an aqueous microdroplets. The observations are not inconsistent, however, because the autoionization of water is enhanced at the air–water interface, producing both more OH^–^ and more H^+^ than in bulk water. Thus, both oxidation and reduction reactions are promoted in water microdroplets.

The travel distance of the microdroplets between the spray source and the MS inlet greatly influences the degree of product conversion, which has been widely reported in many other reactions. This phenomenon seems sensible because of two factors, namely, the increase of reaction time and solvent evaporation, which increased the concentration of reagents.^12^,^14^ In the Dakin reaction of *ortho*-hydroxybenzaldehyde, we varied the distance between the spray emitter and the MS inlet from 1 cm to 11 cm, corresponding to approximate flight times of 100 μs to 13 ms. Because different compounds have different ionization efficiencies, we have corrected the conversion ratios by incorporating the ionization efficiency of each ion. The relative ionization efficiency of intermediate **3** to reactant **1** was 0.19, and that of product **5** to reactant **1** was 0.011. The detailed calculation is shown in the ESI.† As shown in Fig. 2d, as the reaction time increased, the ratio of product **5** to the sum of reagent **1**, intermediate **3**, and product **5** increased gradually from 3.5% to 6.9%, whereas the ratio of intermediate 3 to the same sum decreased from 7.1% to 2.7%. The corresponding mass spectra at the distances of 1 cm and 11 cm are shown in Fig. S3.† Given the microsecond-timescale kinetics, we conclude that the Dakin reaction rate in microdroplets is markedly accelerated compared to the bulk reaction, which typically takes minutes to hours.^15^

**Fig. 3:**
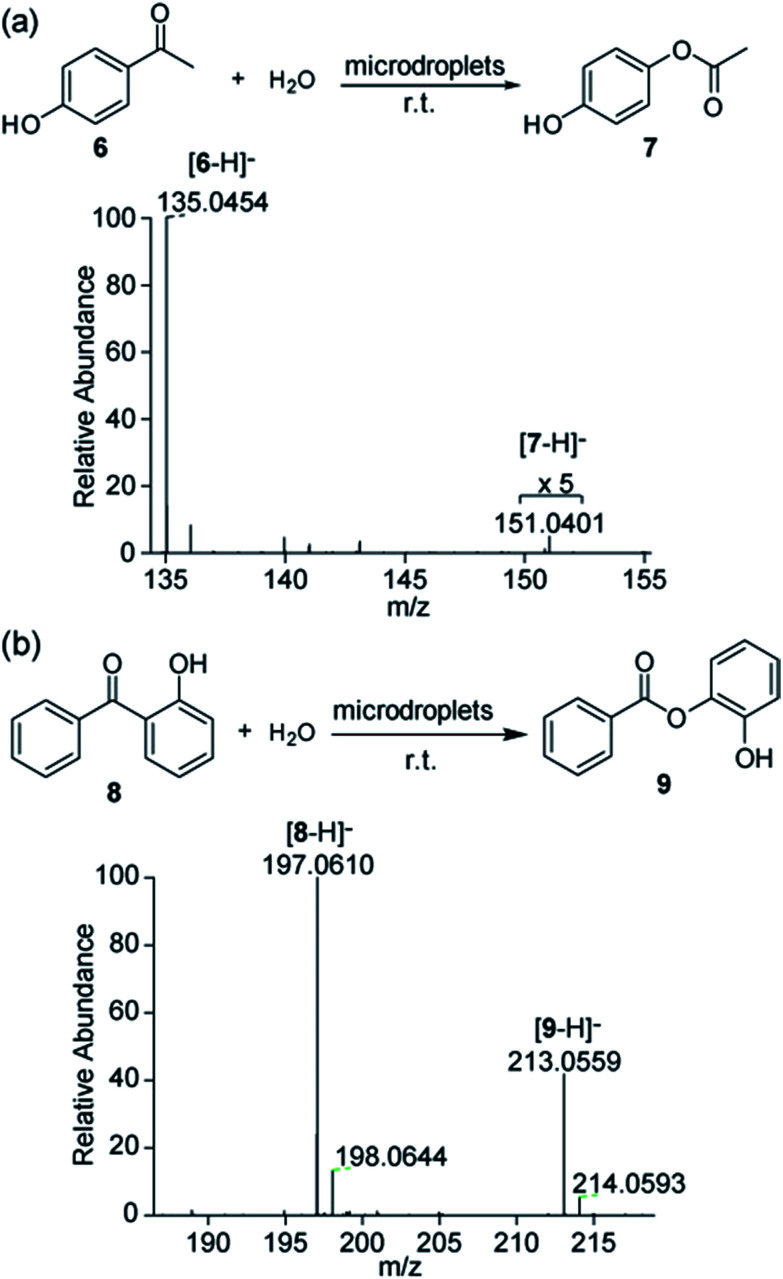
BV reactions in microdroplets of (a) 4-hydroxyacetophenone **6** and (b) 2-hydroxybenzophenone microdroplets without using an acid, base or peroxide and their corresponding mass spectra. For clarity, product **7** is enlarged 5 times.

The BV reaction is another type of oxidation requiring an acid or a base as a catalyst and peroxide as an oxidant. We began our investigation by examining two BV reactions with 4-hydroxyacetophenone **6** and 2-hydroxybenzophenone **8** as the reactants in microdroplets without addition of any catalyst and oxidant. Microdroplets containing **6** and **8** at 100 μM concentration in 1 : 1 water–acetonitrile solution were sprayed separately using nebulizing gas into the MS inlet. Mass spectra were recorded in the negative ion mode. As shown in Fig. 3, the oxidation product, 4-hydroxyphenyl acetate **7** generated from reactant **6**, and product **9** generated from reactant **8** in aqueous microdroplets were detected by the ion signals at *m*/*z* 151.0403 and *m*/*z* 213.0559, respectively. The results indicated the generation of H_2_O_2_ and accumulation of hydrated excess hydronium and hydroxide ions for BV reactions in microdroplets. We also corrected the conversion ratio of the corresponding products in these two BV reactions by measuring the relative ionization efficiencies of the corresponding product to reactant. The calculated values for BV reactions of **6** and **8** were 0.23 and 3.90, see the ESI.† Their corresponding conversion ratios of products **7** and **9** were 2.7% and 3.5%, respectively. Their structures were further confirmed by tandem mass spectrometry using CID (Fig. S4 and S5†). Please note that these BV reactions do not spontaneously occur in bulk water containing a small amount of organic solvent without catalysts and oxidants.

To ensure the critical role of the H_2_O_2_ generated in microdroplets that can induce the BV reaction, 1 eq. and 3 eq. of H_2_O_2_ were added to microdroplets containing reactant **6** to detect by mass spectrometry how the yield of the reaction product changes. When the reaction is carried out in aqueous microdroplets without adding any H_2_O_2_, the yield of product **7** was about 2.7%. However, with the addition of 1 eq. and 3 eq. H_2_O_2_, the yield of product **7** increased to 6.5% and 18.6%, respectively (Table S1†). These findings show that the microdroplets can induce the BV reaction without adding any H_2_O_2_. We also examined the relationship between the concentration of reactant **6** and the conversion yield of product **7** from **6** in microdroplets. The conversion yield increased as the reactant concentration decreased (Fig. S6†), which is consistent with the observation reported in a previous study.^4^ When we decreased the concentration of reactant **6** to 10 nM, this conversion ratio reached 24.6%.

Under bulk conditions, BV reactions are affected by several factors, such as the type of oxidizing agent and reaction conditions, often producing a mixture of phenol and ester.^16^ In an interesting example, Roy *et al.* found that in the BV reaction with sulphuric acid as a catalyst and H_2_O_2_ as an oxidation reagent, only *para*-benzenediol was obtained.^17^ However, we observe exclusively the ester product, but no *para*-benzenediol, when the BV reaction is carried out in aqueous microdroplets without any oxidant and catalyst. These markedly different behaviors between bulk and microdroplet reactions might be caused by the fact that the bulk reactions need longer reaction times (several hours) that allow ester hydrolysis to phenol.

Encouraged by these results, we examined several other BV reactions including ketones in a chain or ring. In all tested reactions, the desired BV oxidation products of individual reactants (cyclohexanone and 3-pentanone) in microdroplets were obtained without adding external catalyst and oxidant (Fig. S7 and S10†).

**Fig. 4:**
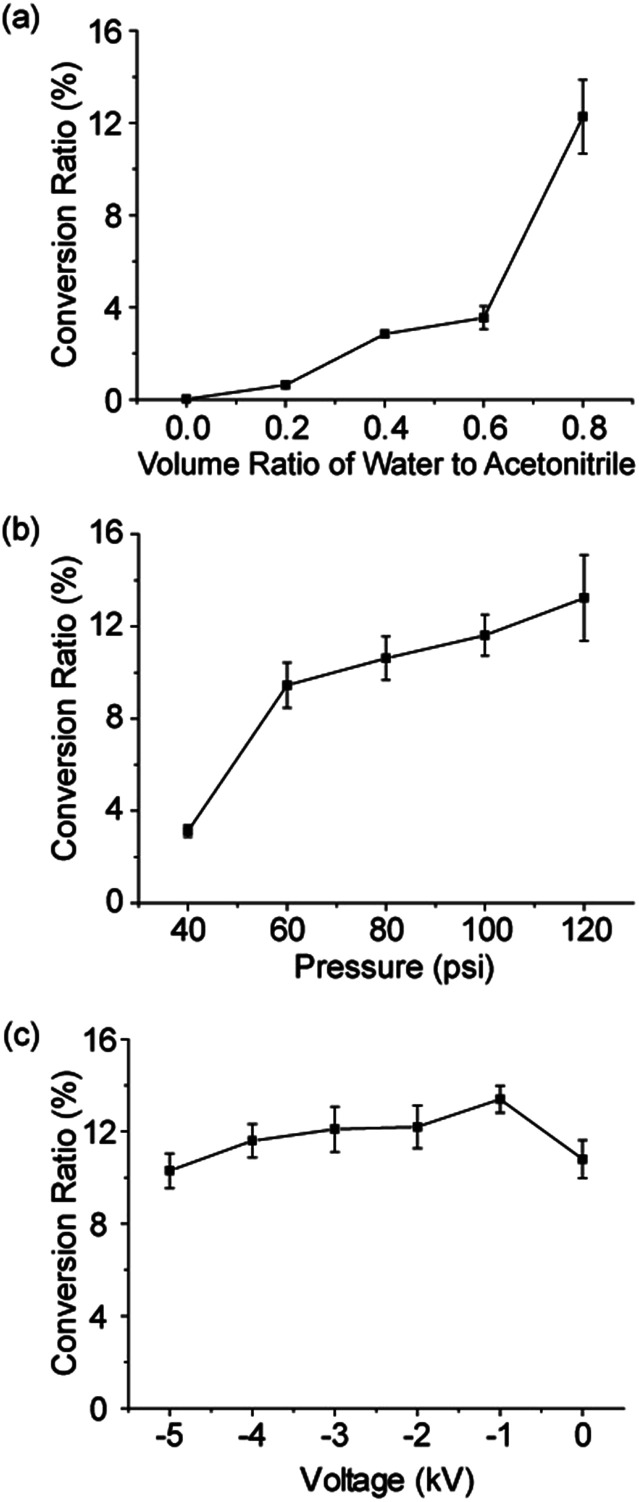
Conversion ratio of product **9** from the BV reaction of reactant **8** in microdroplets with respect to the (a) volume ratio of water to acetonitrile, (b) sheath gas pressure, and (c) voltage applied to the ESI spray source.

As it was suggested by our previous report that H_2_O_2_ was formed by hydroxyl radical recombination,^5^ we investigated the effects of water and organic solvent ratio in microdroplets on the formation of hydroxyl radicals and thus the amount of H_2_O_2_ generated, which will finally influence the product yield of the Dakin and BV reactions. The BV reaction of reactant **8** to form product **9** was tested as an example. Because reactant **8** is not water soluble, we varied the volume ratio of water to ACN (0, 0.2, 0.4, 0.6, and 0.8; v/v) in the microdroplets. The concentration of reactant **8** was kept at 100 μM. As shown in Fig. 4a, the conversion ratio of product **9** increased from 0.03% to 12.5% with increasing concentration of water in the solvent. This result indicates that BV reactions preferentially occur in microdroplets with a high water content. We also noticed that when no water is present in the solvent, there was still a small amount of product **9** generated in microdroplets. The reason might be a trace moisture in the air which led to introduction of water into the microdroplets.

The sharply different behaviors of the Dakin and BV reactions in the bulk from those in aqueous microdroplets emphasize the importance of the droplet size (surface area-to-volume ratio) in driving these two reactions. We further investigated the effect of surface area-to-volume ratio on the product yield in microdroplets for the BV reaction of reactant **8** to form product **9** in aqueous microdroplets. Water/ACN (0.8) was used to dissolve reactant **8** (100 μM) because of the optimal conversion ratio of product **9** under this solvent condition. The reactant solution was then sprayed from the experimental setup with the sheath gas pressure changed in steps of 20 psi from 40 psi to 120 psi to control the droplet size from larger to smaller. The flow rate of the reactant solution was kept at 5 μL min^–1^. As shown in Fig. 4b, we observed that the conversion ratio of product **9** increased gradually from 3.1% to 13.2% with decreasing size of the microdroplets. The improvement of the reaction efficiency in smaller microdroplets suggests the important role played by the surface environment. Smaller microdroplets have higher surface-to-volume ratios so that for the same volume of solution, smaller microdroplets provide more surface sites for chemical reaction.^18^ It is also true that diffusion is more effective in mixing the contents of a microdroplet when it is smaller for a given short reaction time.

Next, we explore the effect of applied voltage on the conversion yield from reactant **8** to product **9** by applying different negative voltages to the spray source (Fig. 4c). When increasing the voltage from –5 to 0 kV, the conversion ratio of product **9** has only a small fluctuation at around 12.0%. These data suggest that the voltage has little or no contribution to this reaction. Possible reasons for the different reaction efficiencies might be related to the varied ionization efficiencies of the reactant and product under different voltages.

## Conclusions

Many previous research studies have reported that the Dakin and BV reactions can be induced by employing H_2_O_2_ as a more environmentally friendly oxidant with only water as an ecologically benign byproduct.^19^ In this work, we have demonstrated that the Dakin and BV reactions can be carried out in microdroplets by employing water as one of the reactants. Compared to the previously reported synthetic methods, water as the reactant and reaction medium has many significant advantages, such as low cost, nonflammability, and environmental compatibility. However, switching from organic solvents to water as a reaction medium is a non-traditional approach, although on-water reactions have received much attention.^20^ As shown in this study, the Dakin and BV reactions can take place in water microdroplets containing a small amount of organic solvent to aid dissolution of the carbonyl compound, but the product yield is rather low. To achieve true green preparative synthesis with these two types of reactions requires further improvements, such as learning how to recycle the sprayed microdroplets, and how to make large quantities of small microdroplets. Our results do demonstrate that microdroplets have a unique microenvironment for inducing chemical reactions, suggesting their potential to be used as powerful microreactors for oxidation and reduction in organic reactions. We also demonstrated that the unique characteristics of aqueous microdroplets have strong potential for practical organic reactions.

## Conflicts of interest

There are no conflicts to declare.

## Supplementary Material

Supplementary information
