# Supplementary material for: Aqueous microdroplets containing only ketones or aldehydes undergo Dakin and Baeyer–Villiger reactions
Source: Chem Sci. 2019 Nov 12;10(48):10974–8. doi: 10.1039/c9sc05112k (PMC7439776; doi:10.1039/c9sc05112k)
Supplement: Supplementary file 1 [file SC-010-C9SC05112K-s001.pdf]

Electronic Supplementary Material (ESI) for Chemical Science.
This journal is © The Royal Society of Chemistry 2019

Aqueous Microdroplets Containing Only Ketones or Aldehydes Undergo Dakin and Baeyer-Villiger Reactions

Dan Gao,‡[ab] Feng Jin,‡[ac] Jae Kyoo Lee,‡[a] Richard N. Zare*[a]

[a]*Department of Chemistry, Stanford University, Stanford, CA 94305.*

[b]*State Key Laboratory of Chemical Oncogenomics, Tsinghua Shenzhen International Graduate School, Tsinghua University, Shenzhen, 518055, China*.

[c]*Shenzhen Deepdrug Information Technology Co. Ltd., Shenzhen, 518052, China*.

Email: zare@stanford.edu

Table of Contents

1. Experimental Procedures Page 2
2. Calculation of the Conversion Efficiency Page 4
3. Supplementary Figures S1-S11 Page 7
4. Supplementary Table S1 Page 19

Experimental Procedures

Chemicals

Ortho-hydroxybenzaldehyde, *para*-hydroxybenzaldehyde, 4-hydroxyacetophenone, cyclohexanone, 2-hydroxyphenyl formate, 7, 4-hydroxyphenyl acetate, 2-hydroxy-benzophenone, 2-hydroxyphenyl benzoate, cyclohexanone, caprolactone, 2-hydro-peroxycyclohexanol, 3-pentanone, ethyl propionate, and propionic acid were purchased from Sigma-Aldrich (St. Louis, MO). HPLC grade water and acetonitrile solvent from Fisher Scientific (Nepean, ON, Canada) were used for all experiments.

Synthesis in Microdroplets and Analysis of Reaction Products

The aqueous solution of the reactant without adding external catalysts or peroxides was injected at the flow rate of 5 μl min^{-1} by a mechanical pump through a syringe to the fused-silica capillary whose output was directed toward a mass spectrometer inlet. A coaxial sheath gas (N_2 at 120 psi) flow around the capillary results in nebulization to form microdroplets. Most reactions were conducted by applying high voltage (-5 kV) to the syringe needle. The temperature of the heated capillary inlet was maintained at approximately 275 ºC and capillary voltage at 44 V. Mass

spectra were all detected by a high-resolution Orbitrap mass spectrometer (LTQ Orbitrap XL Hybrid Ion Trap Orbitrap; Thermo Scientific).

Tandem Mass Spectrometric Analysis

The identification of the observed reaction intermediates and products was carried out by tandem mass spectrometry (MS) using collision-induced dissociation (CID). Fragmentation patterns of reaction intermediates and products were compared with standard samples to confirm the identities of the observed molecules. Both orbitrap and ion trap modes were used for the CID analysis.

Calculation of the Conversion Efficiency

The conversion efficiency for the Dakin reaction of ortho-hydroxybenzaldehyde and BV reaction of 4-hydroxyacetophenone **6** and 2-Hydroxybenzophenone **8** in aqueous microdroplets were calculated by considering the conversion ratios and the relative ionization efficiencies.

For the Dakin reaction of ortho-hydroxybenzaldehyde in aqueous microdroplets, the relative ionization efficiencies between intermediate **3** and ortho-hydroxybenzaldehyde **1**, and the relative ionization efficiencies between product **5** and ortho-hydroxybenzaldehyde **1** were calculated as follows:

$$I = C \times E$$

$$R_1 = \frac{E_3}{E_1}$$

$$R_2 = \frac{E_5}{E_1}$$

where I is the ion intensity of $[M\text{-}H]^-$ in the mass spectrum; C is the concentration of the neutral analytes M; and E is the individual absolute ionization efficiencies of the analytes M, which is calculated according to the method reported in the literature.[1] The absolute ionization efficiency of a compound is dependent on its structure and ionization state as well as the solvent and numerous parameters of the ESI ion source and the mass spectrometer. Several of these parameters are difficult to control, which made it difficult to measure the value for each compound. In contrast to the absolute ionization efficiencies of compounds, their relative ionization efficiencies can be measured quite easily. The values of relative ionization efficiencies can be determined by electrospraying solutions containing analytes at known concentrations

and measuring the intensities of their ions in mass spectrometry. The conversion ratios of the product and intermediate was calculated as follows: E_1, E_3, and E_5 represent the absolute ionization efficiencies of compounds **1**, **3**, and **5**, respectively; R_1 is the relative ionization efficiencies of intermediate **3** to that of ortho-hydroxybenzaldehyde **1**; and R_2 is the relative ionization efficiencies of product **5** to that of ortho-hydroxybenzaldehyde **1**.

$$\frac{C_3}{C_1 + C_3 + C_5} = \frac{I_3/E_3}{I_1/E_1 + I_3/E_3 + I_5/E_5} = \frac{(I_3/E_3) \times E_1}{(I_1/E_1 + I_3/E_3 + I_5/E_5) \times E_1}$$

$$\frac{C_3}{C_1 + C_3 + C_5} = \frac{I_3/R_1}{I_1 + I_3/R_1 + I_5/R_2}$$

$$\text{Conversion ratio in percent of intermediate } \mathbf{3} = \frac{I_3 \times R_2}{I_1 \times R_1 \times R_2 + I_3 \times R_2 + I_5 \times R_1} \times 100\%$$

$$\frac{C_5}{C_1 + C_3 + C_5} = \frac{I_5/E_5}{I_1/E_1 + I_3/E_3 + I_5/E_5} = \frac{(I_5/E_5) \times E_1}{(I_1/E_1 + I_3/E_3 + I_5/E_5) \times E_1}$$

$$\frac{C_5}{C_1 + C_3 + C_5} = \frac{I_5/R_2}{I_1 + I_3/R_1 + I_5/R_2}$$

$$\text{Conversion ratio in percent of product } \mathbf{5} = \frac{I_5 \times R_1}{I_1 \times R_1 \times R_2 + I_3 \times R_2 + I_5 \times R_1} \times 100\%$$

For the BV reaction of 4-hydroxyacetophenone **6** and 2-hydroxybenzophenonein **8**, the relative ionization efficiency between product **7** and reactant **6** and the relative ionization efficiency between product **9** and reactant **8** were calculated from the following formulas:

$$R_3 = \frac{E_7}{E_6}$$

$$R_4 = \frac{E_9}{E_8}$$

$$\frac{C_7}{C_6 + C_7} = \frac{I_7/E_7}{I_6/E_6 + I_7/E_7}$$

$$\text{Conversion ratio in percent of product } \mathbf{7} = \frac{I_7/R_3}{I_6 + I_7/R_3} \times 100\% = \frac{I_7}{I_6 \times R_3 + I_7} \times 100\%$$

$$\text{Conversion ratio in percent of product } \mathbf{9} = \frac{I_9}{I_8 \times R_4 + I_9} \times 100\%$$

Through measuring the same concentrations of the reactant, intermediate, and product by ESI MS under the same MS conditions, their corresponding ion intensities can be obtained for the calculation of the relative ionization efficiency between intermediate and reactant or between product and reactant. The calculated values of R_1, R_2, R_3, and R_4 are 0.19, 0.011, 0.23, and 3.90, respectively.

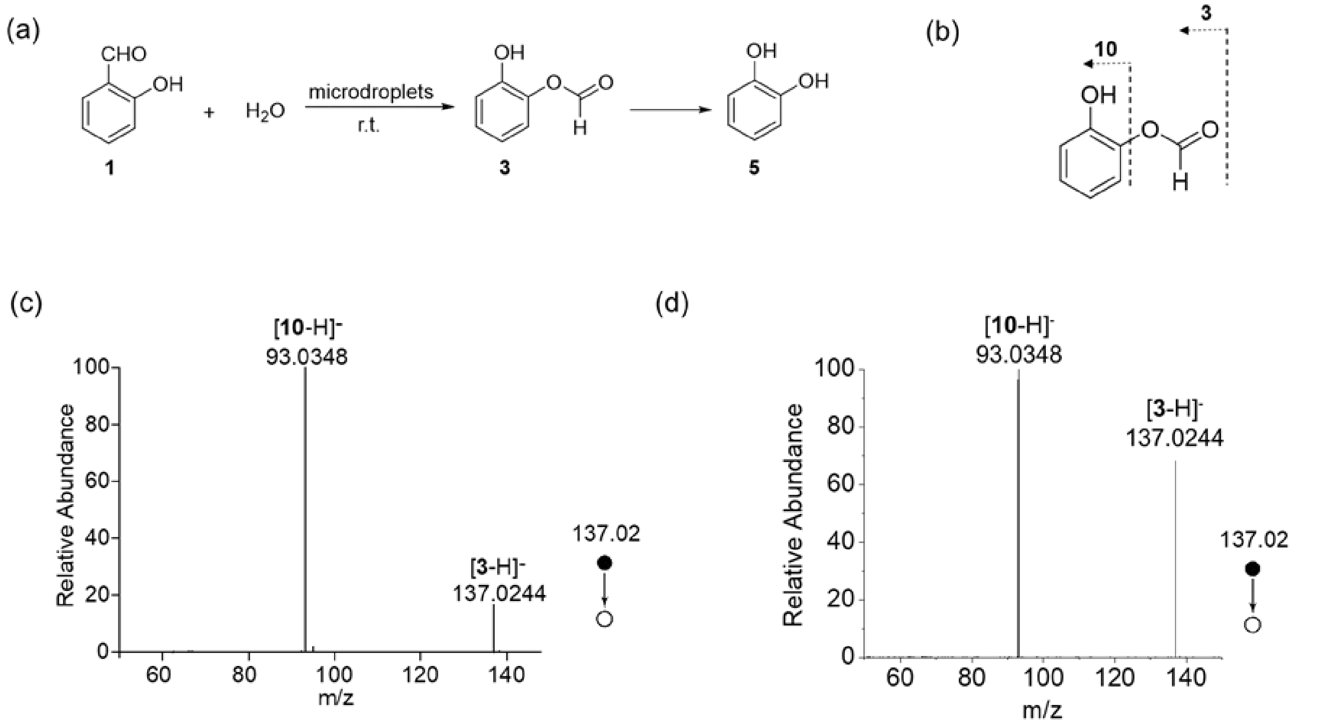

Fig. S1 Tandem MS analysis of the intermediate **3**. (a) The reaction scheme of the Dakin reaction of *ortho*-hydroxybenzaldehyde in water microdroplets to generate a phenyl ester intermeiate **3** and the product **5**, 1,2-dihydroxybenzene**.** (b) The identified structure and fragmentation of **3**. MS/MS spectra of (c) intermediate **3** generated by spraying 100 μM *ortho*-hydroxy-benzaldehyde in microdroplets and (d) standard sample of 100 μM 2-hydroxyphenyl formate.

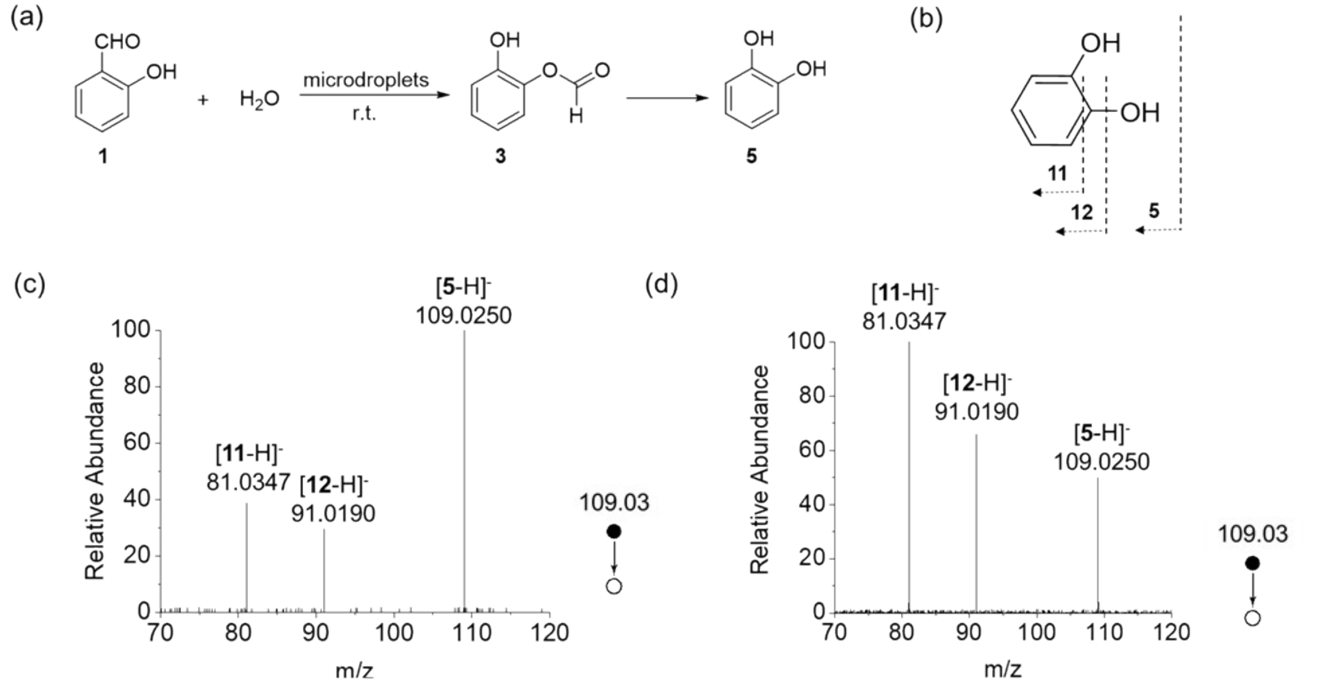

Fig. S2 Tandem MS analysis of the product **5**. (a) The reaction scheme of the Dakin reaction of *ortho*-hydroxybenzaldehyde in water microdroplets to generate a phenyl ester intermediate 3 and the product **5**, 1,2-dihydroxybenzene**.** (b) The identified structure and fragmentation of **5**. MS/MS spectra of (c) the product **5** generated by spraying 100 µM *ortho*-hydroxy-benzaldehyde in microdroplets and (d) standard sample of 100 µM 1,2-dihydroxybenzene.

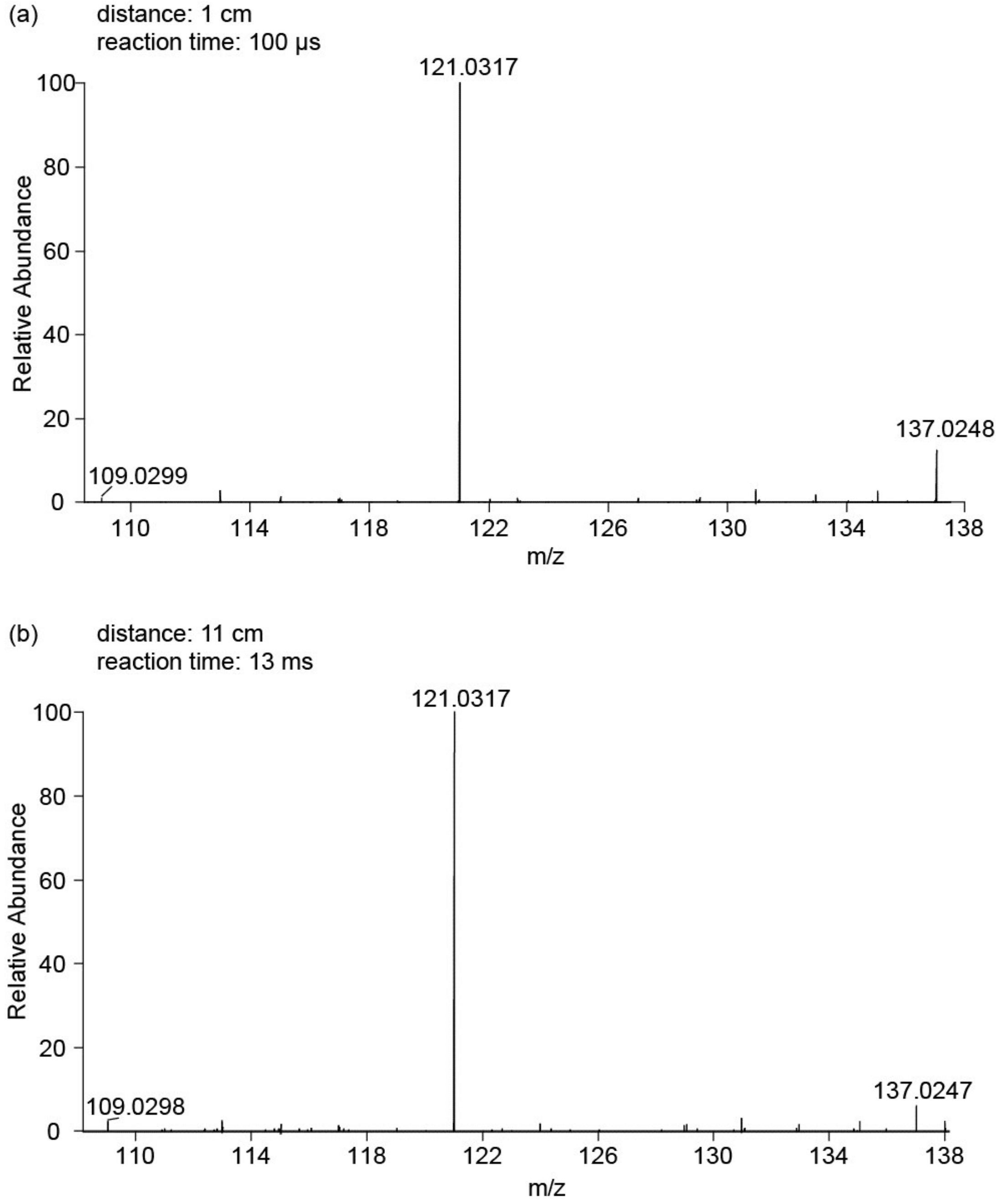

Fig. S3 Mass spectra of the Dakin reaction of 100 µM *ortho*-hydroxybenzaldehyde in water micro-droplets when the distance between the spray emitter to the MS inlet is (a) 1 cm and (b) 11 cm.

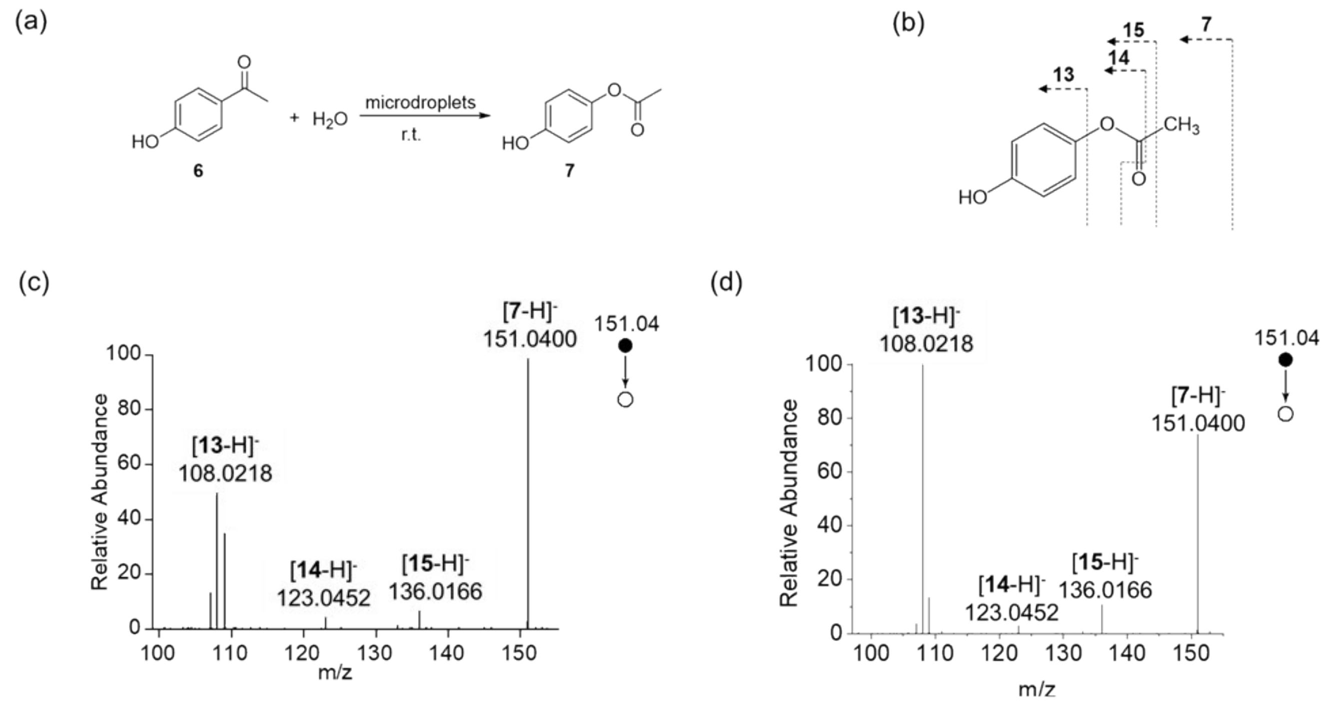

Fig. S4 Tandem MS analysis of the product **7**. (a) The reaction scheme of the BV reaction with 4-hydroxyacetophenone in water microdroplets to generate oxidized product **7**, 4-hydroxyphenyl acetate**.** (b) The identified structure and fragmentation of **7**. (c) MS/MS spectra of the product **7** generated by spraying 100 μM 4-hydroxyacetophenone in water and (d) standard sample of 100 μM 4-hydroxyphenyl acetate.

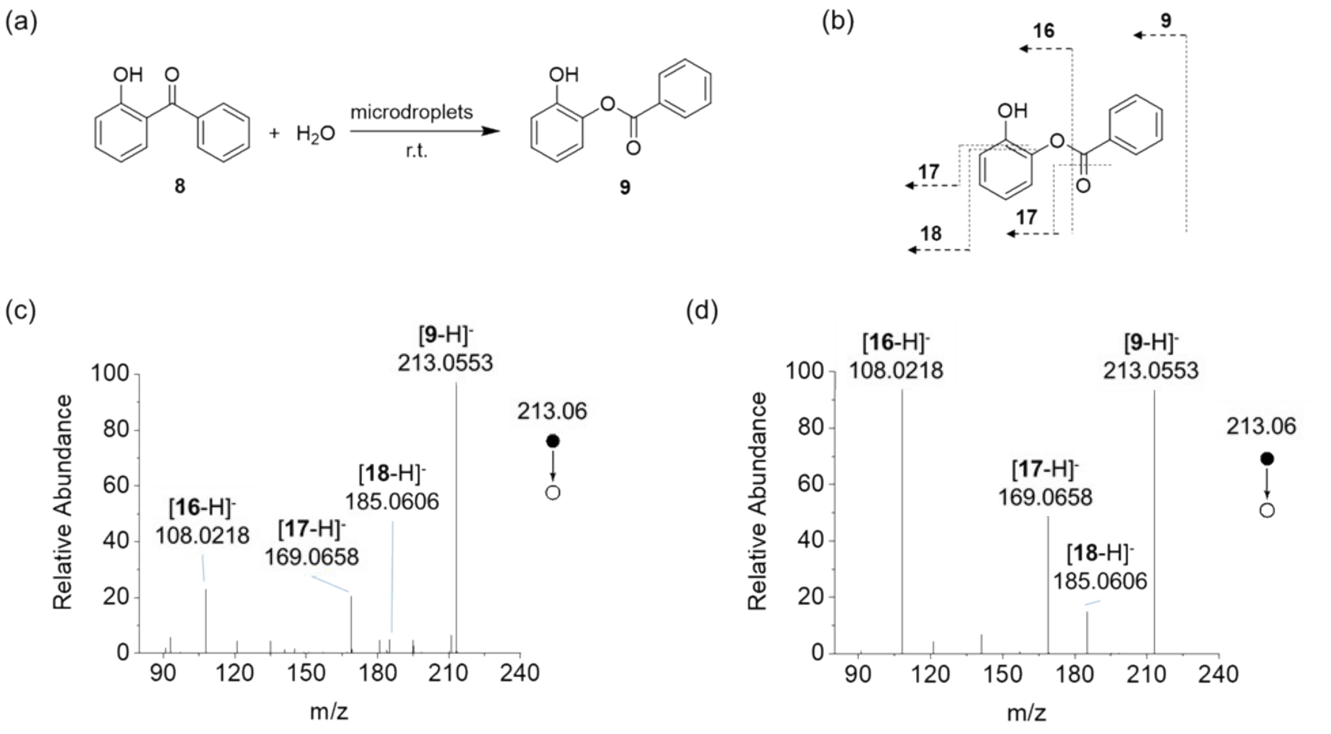

Fig. S5 Tandem MS analysis of the reaction product **9** of the BV reaction of 2-hydroxy-benzophenone **8** to form 2-hydroxyphenyl benzoate **9** in microdroplets. (a) The schematic of the reaction. (b) The identified structure and fragmentation of **9**. (c) MS/MS spectra of the product **9** generated by spraying 100 μM 2-hydroxybenzophenone in microdroplets and (d) standard sample of 100 μM 2-hydroxyphenyl benzoate.

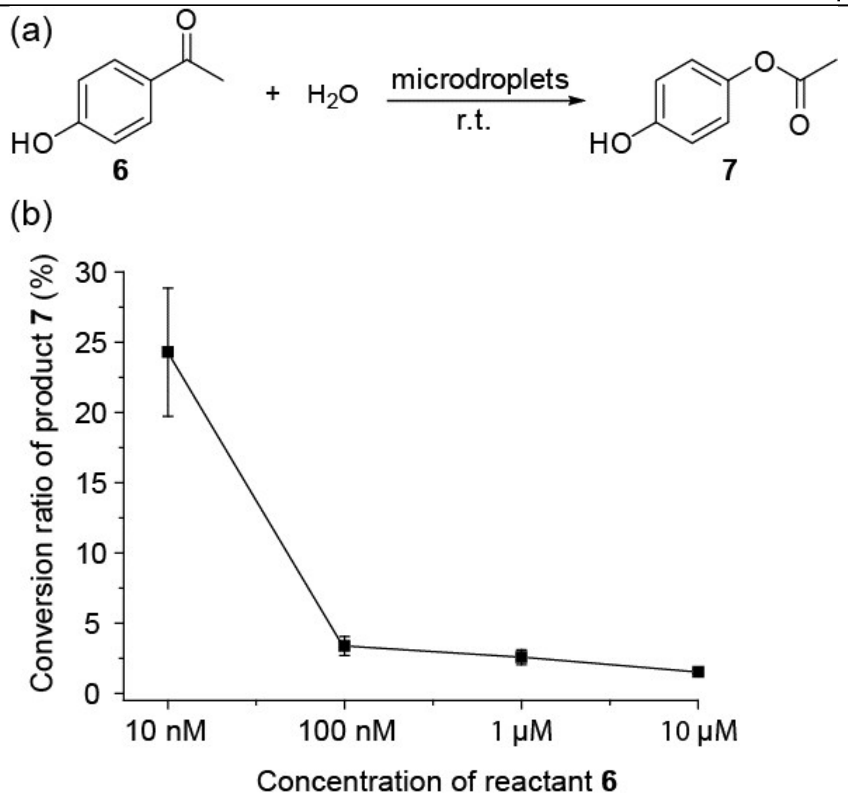

Fig. S6 (a) The BV reaction of 4-hydroxyacetophenone in water microdroplets to generate oxidized product **7**, 4-hydroxyphenyl acetate**.** (b) Dependence of the conversation ratio of product **7** on the concentration of reactant **6.**

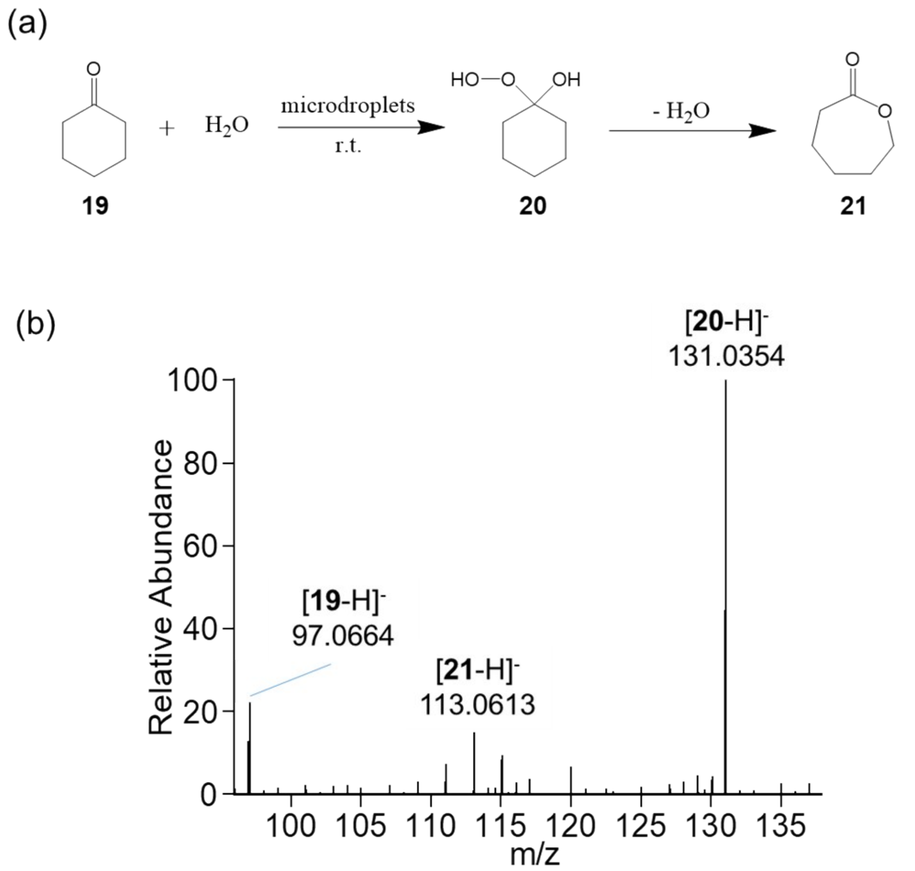

Fig. S7 (a) The BV reaction of cyclohexanone **19** in aqueous microdroplets to form caprolactone **21**. (b) Mass spectrum obtained by spraying 100 μM cyclohexanone **19** in aqueous microdroplets. The observed peaks correspond to reactant, cyclohexanone, (m/z 97.0664, depotonated), intermediate **20** (m/z 131.0354, deprotonated), and product caprolactone (m/z 11.0613).

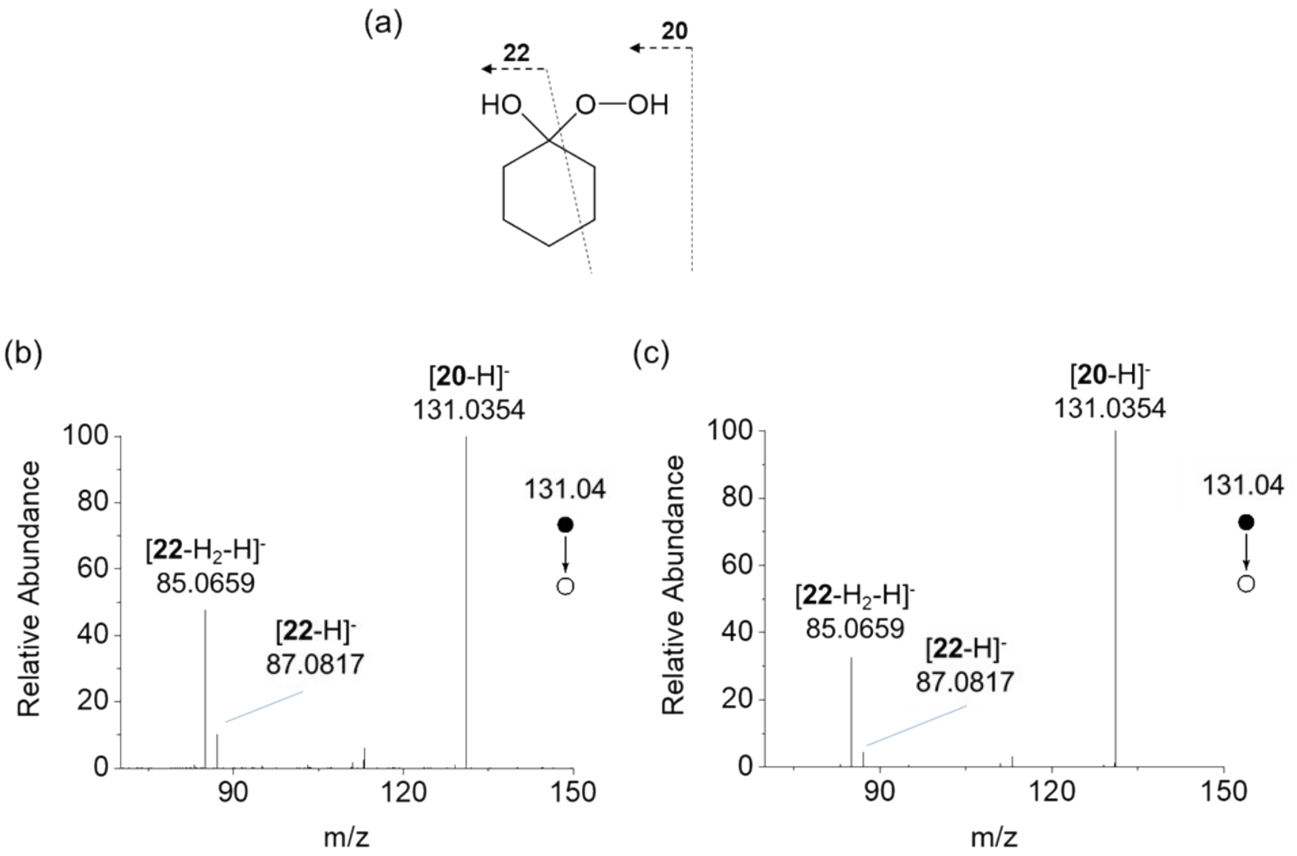

Figure S8. Tandem MS analysis of the reaction imtermediate **20** of the BV reaction of cyclohexanone in microdroplets. (a) The identified structure and fragmentation of **20**. (b and c) MS/MS spectra of the product **20** generated by spraying 100 μM cyclohexanone in microdroplets and standard sample of 100 μM 2-hydroperoxycyclohexanol, respectively.

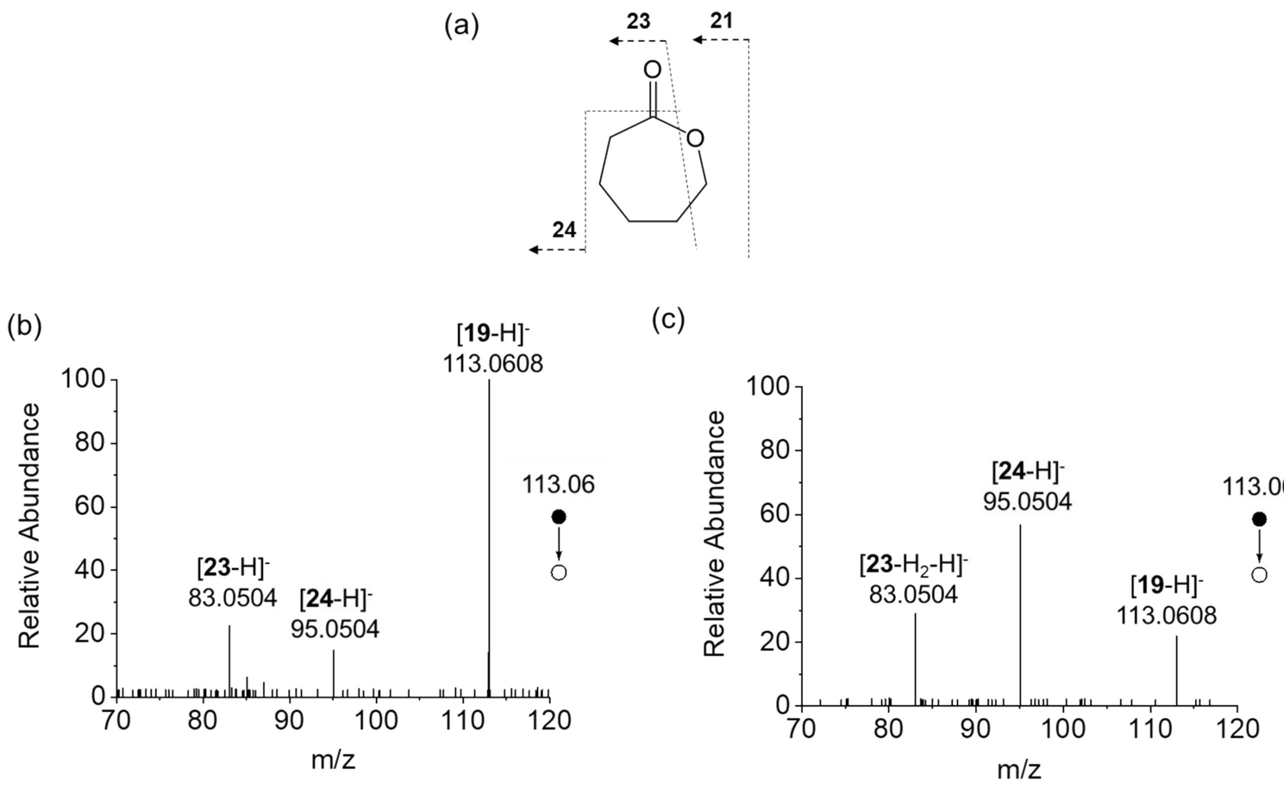

Fig. S9 Tandem MS analysis of the reaction product **21** of the BV reaction of cyclohexanone in microdroplets. (a) The identified structure and fragmentation of **21**. MS/MS spectra of (b) the product **21** generated by spraying 100 μM cyclohexanone in microdroplets and (c) standard sample of 100 μM caprolactone.

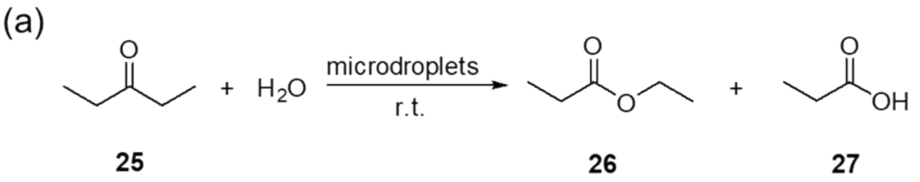

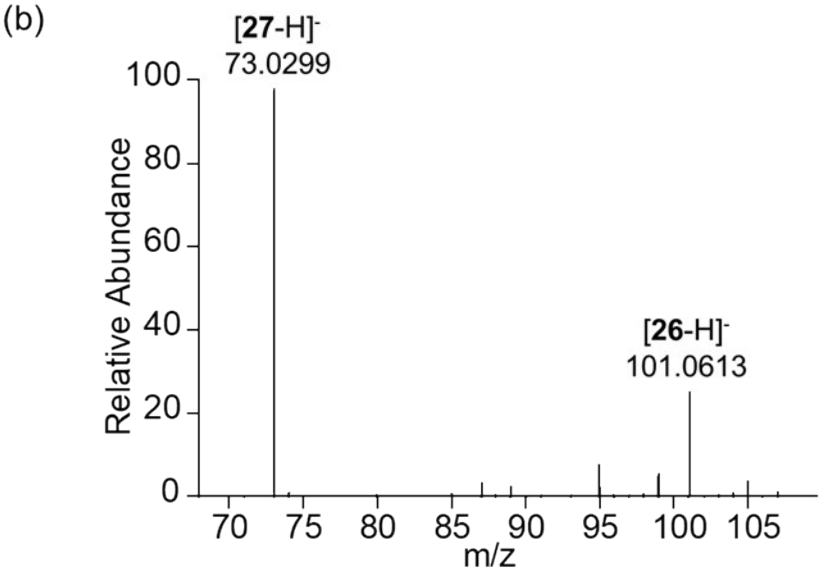

Fig. S10 (a) The BV reaction of 3-pentanone **25** in aqueous microdroplets to form ethyl propionate **26** and propionic acid **27**. (b) Mass spectrum obtained by spraying 100 μM 3-pentanone in aqueous microdroplets. The peaks corresponding reaction products including ethyl propionate (m/z 101.0613 depotonated) and propionic acid (m/z 73.0299, deprotonated) were observed. The reactant was not observed due to the low ionization efficiency of 3-pentanone.

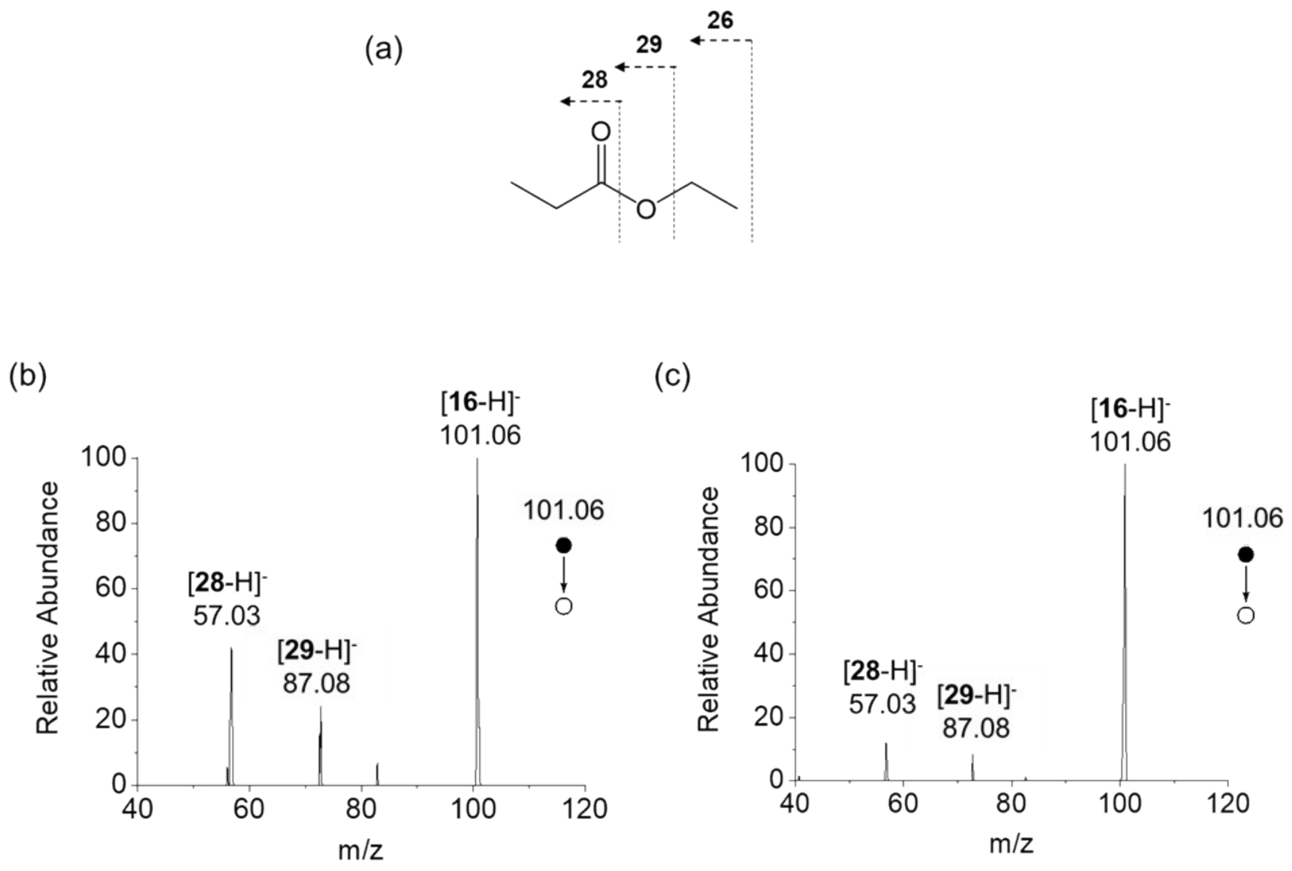

Fig. S11 Tandem MS analysis of the reaction product **26**, ethyl propionate, from the BV reaction of 3-pentanone in microdroplets. (a) The identified structure and fragmentation of **26**. MS/MS spectra of (b) the product **26** generated by spraying 100 μM 3-pentanone in microdroplets and (c) standard sample of 100 μM ethyl propionate.

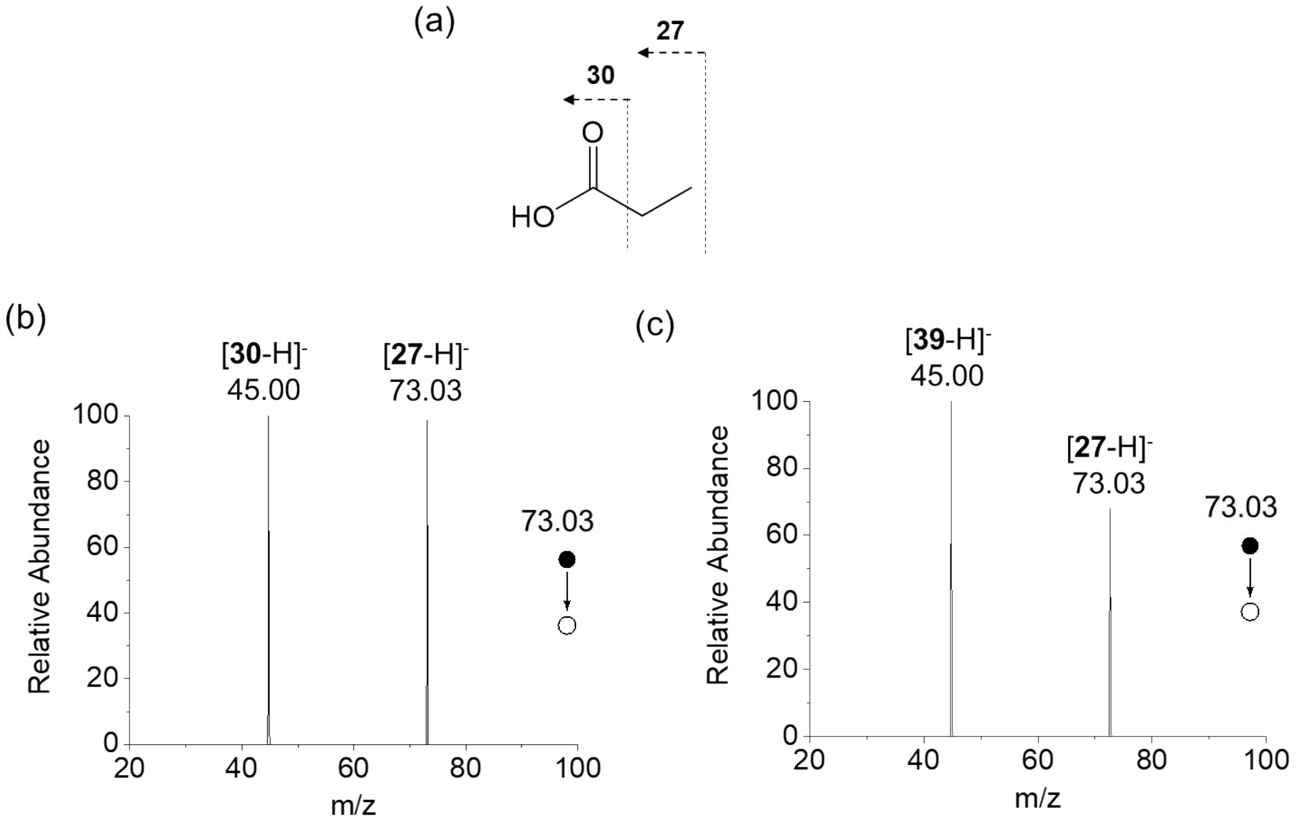

Fig. S12 Tandem MS analysis of the reaction product **27**, propionic acid from the BV reaction of 3-pentanone in microdroplets. (a) The identified structure and fragmentation of **27**. MS/MS spectra of (b) the product **27** generated by spraying 100 μM 3-pentanone in microdroplets and (c) standard sample of 100 μM propionic acid.

Table S1. Conversion ratio of product **7** generated from BV reaction of 100 μM reactant **6** without or with addition of 1 eq. or 3 eq. H_2O_2 in microdroplets.

Reactants in microdroplets	Conversion ratio of product **7** (%)
Reactant **6** with no added H_2O_2	2.7 ± 0.5
Reactant **6** with 1 eq H_2O_2	6.5 ± 0. 8
Reactant **6** with 3 eq H_2O_2	18.6 ± 1.5

Reference

[1] M. Oss, A. Kruve, K. Herodes and I. Leito, *Anal. Chem.* 2010, **82**, 2865.
